# Agreement of claims-based methods for identifying sepsis with clinical criteria in the REasons for Geographic and Racial Differences in Stroke (REGARDS) cohort

**DOI:** 10.1186/s12874-020-00937-9

**Published:** 2020-03-04

**Authors:** John P. Donnelly, Yuling Dai, Lisandro D. Colantonio, Hong Zhao, Monika M. Safford, John W. Baddley, Paul Muntner, Henry E. Wang

**Affiliations:** 1 Department of Learning Health Sciences, University of Michigan Medical School, NCRC Building 14, #G100, G014-130, 2800 Plymouth Rd, Ann Arbor, MI 48109 USA; 2 Institute for Healthcare Policy & Innovation, University of Michigan, Ann Arbor, MI USA; 3 Department of Epidemiology, School of Public Health, University of Alabama at Birmingham, Birmingham, AL USA; 4 Weill Medical College of Cornell University, New York, NY USA; 5 Department of Medicine, Division of Infectious Diseases, University of Alabama at Birmingham, Birmingham, AL USA; 6 Department of Emergency Medicine, The University of Texas Health Science Center at Houston, Houston, TX USA

**Keywords:** Sepsis, Population health, Definitions, Validation

## Abstract

**Background:**

Claims-based algorithms are commonly used to identify sepsis in health services research because the laboratory features required to define clinical criteria may not be available in administrative data.

**Methods:**

We evaluated claims-based sepsis algorithms among adults in the US aged ≥65 years with Medicare health insurance enrolled in the REasons for Geographic And Racial Differences in Stroke (REGARDS) study. Suspected infections from baseline (2003–2007) through December 31, 2012 were analyzed. Two claims-based algorithms were evaluated: (1) infection plus organ dysfunction diagnoses or sepsis diagnoses (Medicare-Implicit/Explicit) and (2) Centers for Medicare and Medicaid Services Severe Sepsis/Septic Shock Measure diagnoses (Medicare-CMS). Three classifications based on clinical criteria were used as standards for comparison: (1) the sepsis-related organ failure assessment (SOFA) score (REGARDS-SOFA), (2) “quick” SOFA (REGARDS-qSOFA), and (3) Centers for Disease Control and Prevention electronic health record criteria (REGARDS-EHR).

**Results:**

There were 2217 suspected infections among 9522 participants included in the current study. The total number of suspected infections classified as sepsis was 468 for Medicare-Implicit/Explicit, 249 for Medicare-CMS, 541 for REGARDS-SOFA, 185 for REGARDS-qSOFA, and 331 for REGARDS-EHR. The overall agreement between Medicare-Implicit/Explicit and REGARDS-SOFA, REGARDS-qSOFA, and REGARDS-EHR was 77, 79, and 81%, respectively, sensitivity was 46, 53, and 57%, and specificity was 87, 82, and 85%. Comparing Medicare-CMS and REGARDS-SOFA, REGARDS-qSOFA, and REGARDS-EHR, agreement was 77, 87, and 85%, respectively, sensitivity was 27, 41, and 36%, and specificity was 94, 92, and 93%. Events meeting the REGARDS-SOFA classification had a lower 90-day mortality rate (140.7 per 100 person-years) compared with the Medicare-CMS (296.1 per 100 person-years), REGARDS-qSOFA (238.6 per 100 person-years), Medicare-Implicit/Explicit (219.4 per 100 person-years), and REGARDS-EHR classifications (201.8 per 100 person-years).

**Conclusion:**

Claims-based sepsis algorithms have high agreement and specificity but low sensitivity when compared with clinical criteria. Both claims-based algorithms identified a patient population with similar 90-day mortality rates as compared with classifications based on qSOFA and EHR criteria but higher mortality relative to SOFA criteria.

## Background

Sepsis, defined as organ dysfunction resulting from a dysregulated host response to infection, is a major cause of death worldwide. In the United States (US) alone, there are over 1.5 million hospitalizations, 850,000 Emergency Department (ED) visits, and 250,000 deaths annually attributable to sepsis [1–3]. Population-based estimates of sepsis incidence rates, mortality rates, and case fatality have been shown to vary across methods for defining sepsis [4–6]. The Third International Consensus Definitions for Sepsis (Sepsis-3) guidelines, published in 2016, defined sepsis by the presence of an infection with acute organ dysfunction based on systems included in the sepsis-related organ failure assessment (SOFA) score [7]. The Sepsis-3 task force also derived a novel severity score based on “quick” SOFA (qSOFA) criteria (i.e., low blood pressure, high respiratory rate, and altered mental status) [7]. An alternative approach, based on clinical data available in electronic health records (EHR), has also been proposed [4, 8]. When clinical data are not available, discharge diagnosis codes have been used to define sepsis. The Centers for Medicare and Medicaid Services (CMS) Severe Sepsis/Septic Shock Core Measure defines sepsis based upon discharge diagnosis codes for sepsis or septicemia [9, 10]. In addition, a modified version of the algorithm presented by Angus, et al., is one of the most widely utilized approaches for identifying sepsis in administrative claims, and is defined by the concurrent presence of discharge diagnosis codes for infection and acute organ dysfunction (the “implicit” method) or an explicit code for sepsis [11].

The use of discharge diagnoses to identify sepsis in claims data facilitates the analysis of large databases but may not identify lower acuity sepsis cases and those not reported by medical coders. Clinical criteria offer greater certainty of sepsis classification but require either chart abstraction or the implementation of EHR algorithms to obtain laboratory measurements. Several prior studies have compared claims-based approaches for defining sepsis with approaches based on older systemic inflammatory response syndrome (SIRS) criteria [12–14]. However, few prior studies have compared claims-based algorithms with contemporary clinical criteria and there is a paucity of data from large population-based cohorts [4, 15]. Although CMS and other regulatory bodies have transitioned to use of International Classification of Diseases, 10th revision codes (ICD-10) many existing claims databases still require use of International Classification of Diseases, 9th revision codes (ICD-9).

Using data from the REasons for Geographic and Racial Differences in Stroke (REGARDS) study cohort linked with Medicare claims, we assessed agreement between claims-based approaches and clinical criteria for identifying sepsis. We also compared baseline participant characteristics and mortality rates across clinical criteria and claims-based approaches.

## Methods

### Study population

The REGARDS study enrolled 30,239 white and black adults aged ≥45 years from the 48 contiguous US states and the District of Columbia between 2003 and 2007 [16]. At baseline, all participants completed a phone interview followed by an in-home examination. Data from REGARDS study participants have been linked with Medicare claims [17, 18]. For the current analysis, we included REGARDS study participants who had their data linked to Medicare claims and had Medicare Parts A (inpatient services) and B (outpatient services) coverage, without Part C coverage (Medicare managed care) and who were 65 years of age or older on the date of their REGARDS baseline examination. Medicare coverage was defined using data from the beneficiary enrollment file. The primary analyses were further restricted to study participants with suspected infection events identified and adjudicated through REGARDS study procedures as defined below.

Consistent with the REGARDS study protocol approved by the University of Alabama at Birmingham Institutional Review Board and the institutional review boards governing human subjects research at participating institutions, participants provided verbal consent before completing the baseline telephone interview and written informed consent before completing the in-home examination. The consent form included permission to merge participant data with Medicare claims. For this analysis, we conducted an observational study using data from REGARDS. The study was approved by the University of Alabama at Birmingham Institutional Review Board with waiver of the requirement for additional informed consent (approval number X090531004). Additional information related to ethics approval, including example consent forms, is available at the REGARDS study website (http://www.regardsstudy.org).

### REGARDS adjudicated infection events and clinical criteria for Sepsis

Study personnel contacted participants at 6-month intervals by telephone to identify the date, location, and attributed reason for hospitalizations or ED visits. Example baseline and 6-month follow-up data collection forms are available at the REGARDS study website (http://www.regardsstudy.org). We identified hospitalizations or ED visits for which the participant reported an infection as the main cause or reason for admission (defined hereinafter as suspected infection events) [11] (Additional file 1: Appendix A of the Online Methods Supplement). All relevant medical records were retrieved for suspected infection events and two trained research assistants independently reviewed medical records to confirm infection as a primary reason for hospitalization based on standardized abstraction criteria [11] (Additional file 1: Appendix B of the Online Methods Supplement). Disagreements between the two reviewers were resolved by a physician (HEW). We included events from February 5, 2003, through December 31, 2012, and a review of 1349 records indicated excellent agreement between reviewers for the presence of infection (kappa = 0.92). We excluded events occurring after a participant discontinued Medicare Part A or B coverage or initiated Medicare Part C coverage as these events are not expected to be present in claims.

To define clinical criteria, we used physiologic and laboratory measurements from the initial 28 h of the confirmed infection hospitalizations. We examined three classifications based on clinical criteria, REGARDS-SOFA, REGARDS-qSOFA, and REGARDS-EHR. REGARDS-SOFA sepsis events were classified as hospitalizations with infection and acute organ dysfunction, defined as ≥2 SOFA points across all organ systems (respiratory, cardiovascular, renal, hematological, hepatic, and neurological) [19]. REGARDS-qSOFA infection events were classified based on the presence of ≥2 qSOFA criteria, including altered mentation (Glasgow coma score < 15 or non-alert per the Alert, Voice, Pain, Unresponsive scale), systolic blood pressure ≤ 100 mmHg, or respiratory rate ≥ 22 breaths per minute [20]. REGARDS-EHR events were identified using a modified version of an EHR definition adopted for surveillance by the Centers for Disease Control and Prevention, with sepsis events classified as infection with acute organ dysfunction (vasopressor use, mechanical ventilation, serum creatinine ≥2.0 mg/dL, total bilirubin ≥2.0 mg/dL, platelet count < 100 cells/μL, or serum lactate ≥2.0 mmol/L) [4]. Consistent with prior studies, missing values for criteria were considered normal [20]. We did not evaluate changes in measurements from baseline, as only the worst values were collected and the only pre-hospitalization measures that were available were taken at baseline enrollment.

### Claims-based Sepsis events

We defined episodes of care using Medicare inpatient, outpatient, or carrier claims between 2003 and 2012 with an ICD-9 diagnosis code for infection, organ dysfunction, or sepsis (Additional file 1: Appendix C and Appendix D of the Online Methods Supplement). Claims were combined into the same episode of care if the discharge date from one inpatient claim was on the admission date or the day before a subsequent inpatient claim. Using modified taxonomies published by Angus, et al., we classified episodes of care with ICD-9 discharge diagnosis codes for both infection and organ dysfunction or an explicit ICD-9 discharge diagnosis code for severe sepsis or septic shock as “Medicare-Implicit/Explicit” sepsis [11]. We also defined episodes of care with ICD-9 codes for sepsis or septicemia outlined in the CMS sepsis measure as “Medicare-CMS” sepsis [9].

Suspected infections detected through REGARDS study procedures and episodes of care defined using Medicare claims were considered the same event if the date of admission identified in the REGARDS study was between the episode of care start and end dates, inclusive, in Medicare claims. In a sensitivity analysis, we investigated episodes of care in Medicare detected as infection events through REGARDS study procedures for which infection was identified as a primary reason for hospitalization by chart abstractors. This approach allowed us to have greater certainty that participants presented with infection and excluded infections which were possibly hospital-acquired.

### Participant baseline characteristics

Baseline participant characteristics were determined upon enrollment into the REGARDS study. A description of variable definitions is provided in Additional file 1: Appendix E of the Online Methods Supplement. Demographic characteristics included gender, race, and self-reported annual household income and education (maximum level achieved). We also identified several behaviors, including smoking and alcohol use [21]. Medical conditions included atrial fibrillation, chronic kidney disease (CKD), diabetes, dyslipidemia, hypertension, lung disease, myocardial infarction (MI), obesity, and stroke [22, 23]. Using baseline characteristics, we calculated a Sepsis Risk Score for each participant [24]. Year of hospitalization and age at the time of hospitalization were also determined.

### Mortality

We defined 90-day mortality relative to the admission date using deaths identified through the Medicare beneficiary summary file. In-hospital deaths were included in the 90-day mortality rate. All deaths recorded in the Medicare beneficiary summary file were also detected through REGARDS study procedures and death dates were confirmed by REGARDS personnel using death certificates, autopsy reports, and medical records.

### Statistical analysis

We calculated participant characteristics for suspected infection events which were classified as sepsis and non-sepsis events based on clinical criteria (i.e., REGARDS-SOFA, REGARDS-qSOFA, and REGARDS-EHR) and Medicare claims (i.e., Medicare-Implicit/Explicit and Medicare-CMS) as means and standard deviations for continuous variables and percentages for categorical variables.

We estimated the agreement of each Medicare sepsis definition (Medicare-Implicit/Explicit and Medicare-CMS) with each classification from the REGARDS study (REGARDS-SOFA, REGARDS-qSOFA, and REGARDS-EHR), constructing spine plots to graphically depict concordant and discordant cells. We calculated kappa statistics and observed agreement as well as sensitivity, specificity, positive predictive value (PPV), and negative predictive value (NPV) for each Medicare-sepsis algorithm, using REGARDS-SOFA, REGARDS-qSOFA, or REGARDS-EHR, one at a time, as the standard [25–29]. As multiple events for the same participant were included, we estimated confidence intervals (CIs) for all measures using bootstrapping with 1000 resamples and bias-correction, accounting for clustering by participant identifier. We repeated these calculations restricting the analysis to (1) events occurring in the more recent years under study (2009–2012), and (2) episodes of care in Medicare claims detected as infection events through REGARDS study procedures for which infection was a primary reason for hospitalization.

We calculated 90-day mortality rates for participants with each definition and classification, with follow-up time starting from the admission date for the REGARDS infection event. For calculating the 90-day mortality rate, participants were censored upon the loss of Medicare Part A or B coverage or initiation of Medicare Part C coverage, loss to follow-up in the REGARDS study, or at 90 days from admission, whichever occurred first. In order to compare mortality rates for participants with and without specified criteria, we assessed the associations of the REGARDS study classifications and claims-based definitions with 90-day mortality by comparing concordant and discordant cells. We also fit Cox proportional hazards models with events meeting neither classification as the reference group, adjusting for participant characteristics and accounting for clustering by participant identifier. We confirmed that the proportional hazards assumption was met using Schoenfeld residuals.

We conducted all analyses using Stata 13.1 (StataCorp, College Station, Texas).

## Results

### Event incidence and population characteristics

**Fig. 1:**
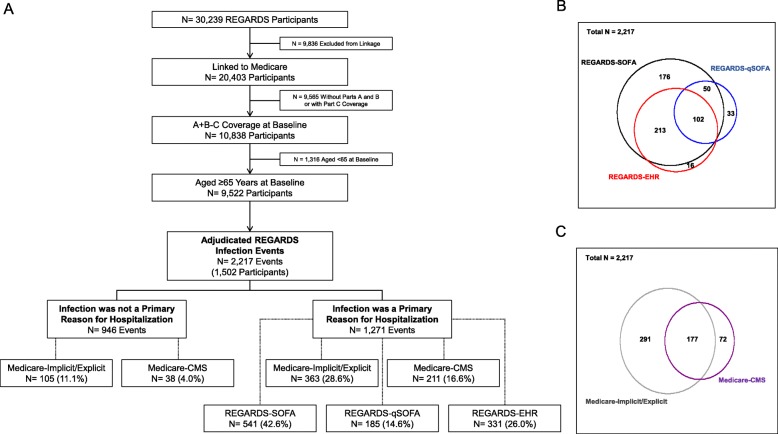
Study population flowchart and proportional Venn diagrams showing overlap of criteria. Legend: Identification methods not mutually exclusive, events can belong to multiple groups. Implicit/Explicit ICD-9 sepsis defined based on taxonomies of infection and organ dysfunction codes proposed by Angus, et al. in addition to explicit sepsis codes. CMS ICD-9 sepsis defined based on list of codes used in the CMS measure. REGARDS-EHR defined as infection event meeting modified EHR criteria proposed by Rhee, et al.^4^ REGARDS-SOFA defined as infection event with ≥2 SOFA points across all organ systems (respiratory, cardiovascular, renal, hematological, hepatic, and neurological). REGARDS-qSOFA defined as infection events meeting ≥2 qSOFA criteria. Panel A) Population flowchart; Panel B) Venn diagram showing overlap between REGARDS-SOFA, REGARDS-qSOFA, and REGARDS-EHR; Panel C) Venn diagram showing overlap between Medicare-Implicit/Explicit and Medicare-CMS. Population for proportional Venn diagrams include all adjudicated hospitalizations, represented by the overall square area (Panel A: *N* = 1627 not meeting any classification; Panel B: *N* = 1677 not meeting either definition). REGARDS = REasons for Geographic and Racial Differences in Stroke study; A + B-C = participants with Medicare Parts A and B without Part C (managed care); SOFA = sepsis-related organ failure assessment; qSOFA = “quick” sepsis-related organ failure assessment; EHR = electronic health record; CMS = Centers for Medicare and Medicaid Services; ICD-9 = International Classification of Diseases, Ninth Revision

After excluding REGARDS study participants not linked to Medicare, without Parts A and B coverage, with Medicare Part C coverage, and those aged < 65 years at baseline, the final analytic cohort included 9522 participants (Fig. 1a). Among these participants, there were 2217 suspected infection events detected through REGARDS study procedures. Most infection events that met the REGARDS-qSOFA and REGARDS-EHR classifications also met REGARDS-SOFA criteria (82.2 and 95.2%, respectively), whereas a substantial proportion of REGARDS-SOFA events did not meet the other criteria (32.5%) (Fig. 1b). Most Medicare-CMS events met the Medicare-Implicit/Explicit definition (71.1%), while most Medicare-Implicit/Explicit events did not meet the Medicare-CMS definition (62.2%) (Fig. 1c). Among all suspected infection events, REGARDS-SOFA was the most commonly met classification (24.4%), followed by Medicare-Implicit/Explicit (21.1%) and REGARDS-EHR (14.9%). Medicare-CMS and REGARDS-qSOFA criteria were met for 11.2 and 8.3% of suspected infection events, respectively. Compared to suspected infection events without sepsis by any classification (columns 1 and 2), a higher proportion of those with sepsis were in male, older adults, and adults with more comorbidities and a higher sepsis risk score at baseline. (Table 1) The percent of infection events meeting criteria increased over the study period for Medicare-Implicit/Explicit, REGARDS-SOFA, and REGARDS-EHR, but remained similar for other classifications (Additional file 2: Appendix F of the Online Results Supplement).

**Table 1:** Participant characteristics for suspected infection events by sepsis identification method (*N* = 2217)

Characteristic	No Infection as Primary Reason / No Sepsis	Infection as Primary Reason / No Sepsis	Claims-based ICD-9 Methods		Abstracted Clinical Criteria		
			Medicare-Imp/Exp	Medicare- CMS	REGARDS-SOFA	REGARDS-qSOFA	REGARDS-EHR
**Total N**	*N* = 834	*N* = 557	*N* = 468	*N* = 249	*N* = 541	*N* = 185	*N* = 331
**Age (yrs) (Mean/SD)**	77.1 (6.6)	76.1 (6.2)	78.4 (6.3)	78.0 (6.4)	78.3 (6.8)	77.8 (6.5)	78.0 (6.5)
**Gender (%)**							
Male	50.5	46.3	60.7	59.8	66.9	66.0	66.5
Female	49.5	53.7	39.3	40.2	33.1	34.1	33.5
**Race (%)**							
White	76.4	79.2	78.4	74.3	77.8	81.1	75.8
Black	23.6	20.8	21.6	25.7	22.2	18.9	24.2
Education (%)*							
< High School	12.8	15.1	16.3	14.1	15.2	17.3	16.9
High School	27.7	26.8	29.3	31.3	29.2	31.4	28.7
Some College	27.3	29.8	26.8	29.3	28.1	31.4	27.2
College or More	32.1	28.4	27.6	25.3	27.5	20.0	27.2
**Income (%)**							
< $20 k	19.7	19.4	22.0	21.3	19.2	19.5	17.2
$20-$34 k	28.4	29.6	29.1	30.5	28.7	30.3	27.5
$35-$74 k	31.8	29.1	32.1	30.1	33.6	27.6	35.4
≥ $75 k	6.7	7.9	6.8	4.8	7.2	7.0	8.5
Not Available	13.4	14.0	10.0	13.3	11.3	15.7	11.5
Smoking (%)**							
Never	38.3	43.4	33.0	37.0	35.9	29.4	33.3
Past	51.4	48.7	56.5	52.2	53.7	54.4	55.2
Current	10.2	7.9	10.5	10.8	10.4	16.3	11.5
**Alcohol Use (%)**†							
None	64.7	69.5	68.0	Supp	69.3	Supp	71.8
Moderate	31.4	27.8	28.1	26.0	27.3	27.9	23.9
Heavy	3.9	2.8	3.9	*N* < 11(Supp)	3.4	N < 11(Supp)	4.3
**Comorbidities (%)**							
Atrial Fibrillation	15.5	13.6	17.1	20.1	17.7	15.7	17.8
Lung Disease	18.4	16.9	25.0	20.5	19.2	20.0	22.1
Chronic Kidney Disease‡	23.3	20.5	39.7	43.8	43.6	36.2	53.5
Stroke	11.3	9.5	15.4	16.9	11.8	14.1	12.1
Myocardial Infarction	19.4	22.4	26.7	26.1	26.8	27.6	27.8
Hypertension	63.2	63.0	68.8	72.3	71.0	68.1	71.3
Dyslipidemia	62.0	63.4	64.3	68.7	69.3	71.4	71.3
Diabetes	23.0	24.6	34.0	36.1	35.3	36.2	39.3
Obesity	50.5	56.6	52.4	54.2	53.4	55.7	55.3
**Comorbidities (Mean/SD)**	2.9 (1.5)	2.9 (1.5)	3.4 (1.5)	3.6 (1.5)	3.5 (1.6)	3.4 (1.4)	3.7 (1.5)
**Risk Score (%)**							
Low (0–7 Points)	22.7	26.0	9.6	6.8	12.4	13.0	7.6
Middle (8–12 Points)	36.0	32.1	31.6	34.5	27.0	28.1	25.7
High (13–32 Points)	41.4	41.8	58.8	58.6	60.6	58.9	66.8

Total N = 2217 adjudicated hospitalizations. Identification methods not mutually exclusive, events can belong to multiple groups. Implicit/Explicit ICD-9 sepsis defined based on taxonomies of infection and organ dysfunction codes proposed by Angus, et al. CMS ICD-9 sepsis defined based on list of codes used in the CMS SEP-1 measure. REGARDS-EHR defined as infection event meeting modified EHR criteria proposed by Rhee, et al. REGARDS-SOFA defined as infection event with ≥2 SOFA points. REGARDS-qSOFA defined as infection event meeting ≥2 qSOFA criteria. *1 missing. **6 missing. †38 missing. ‡Chronic kidney disease defined using creatinine values and the CKD-EPI eq. N < 11(Supp) indicates a cell size less than 11, which is suppressed per our data use agreement. SOFA = sepsis-related organ failure assessment; qSOFA = “quick” sepsis-related organ failure assessment; EHR = electronic health record. SD = standard deviation; CMS = Centers for Medicare and Medicaid Services; ICD-9 = International Classification of Diseases, Ninth Revision

### Agreement between claims-based methods and clinical criteria

**Fig. 2:**
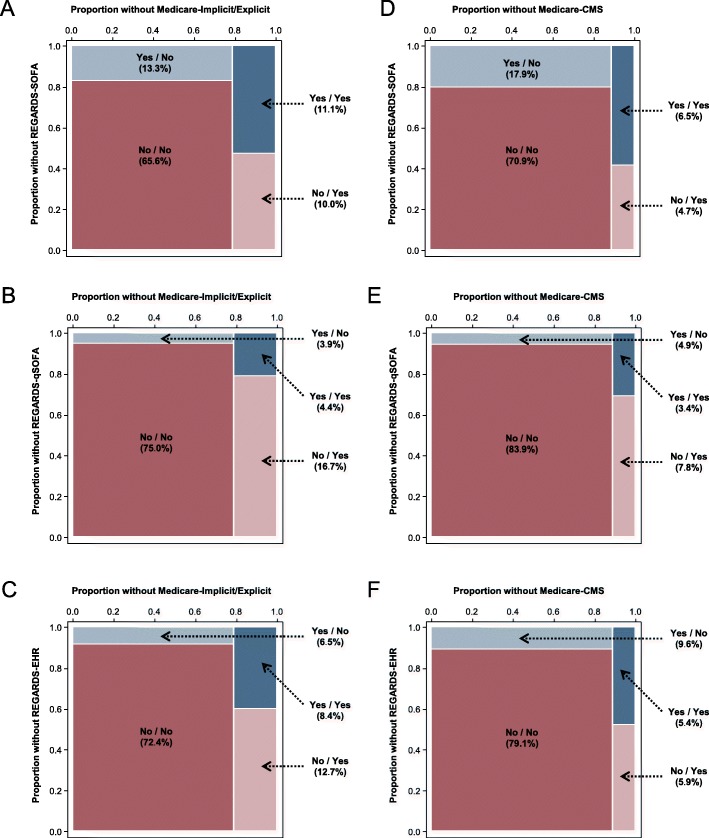
Spine plots showing agreement between claims-based methods for sepsis identification and abstracted clinical criteria (*N* = 2217). Legend: Total N = 2217 adjudicated hospitalizations. Implicit/Explicit ICD-9 sepsis defined based on taxonomies of infection and organ dysfunction codes proposed by Angus, et al. in addition to explicit sepsis codes. CMS ICD-9 sepsis defined based on list of codes used in the CMS measure. REGARDS-EHR defined as infection event meeting modified EHR criteria proposed by Rhee, et al.^4^ REGARDS-SOFA defined as infection event with ≥2 SOFA points across all organ systems (respiratory, cardiovascular, renal, hematological, hepatic, and neurological). REGARDS-qSOFA defined as infection events meeting ≥2 qSOFA criteria. Light blue cells indicate events with REGARDS and without Medicare. Light red cells indicate events without REGARDS and with Medicare. Panels A-C) Concordance between Medicare-Implicit/Explicit and abstracted clinical criteria (REGARDS-SOFA, REGARDS-qSOFA, and REGARDS-EHR). Panels D-F) Concordance between Medicare-CMS and abstracted clinical criteria (REGARDS-SOFA, REGARDS-qSOFA, and REGARDS-EHR). Rectangle areas represent cell proportions. Cell percentages shown in rectangles or next to arrows. SOFA = sepsis-related organ failure assessment; qSOFA = “quick” sepsis-related organ failure assessment; EHR = electronic health record. CMS = Centers for Medicare and Medicaid Services; ICD-9 = International Classification of Diseases, Ninth Revision

**Fig. 3:**
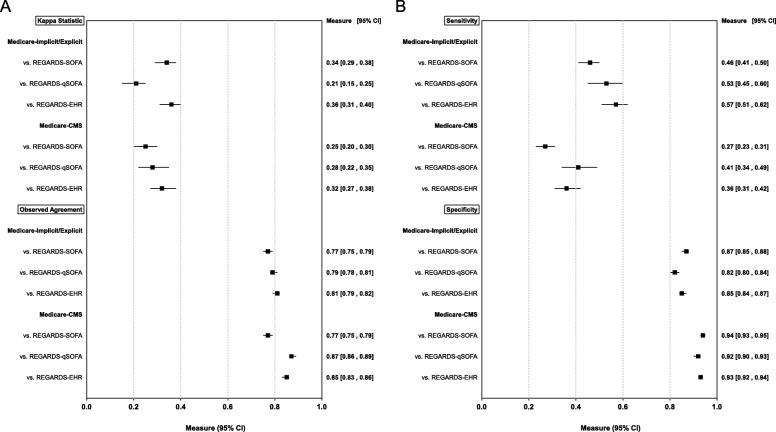
Measures of agreement and validity between claims-based methods for sepsis identification and sepsis definitions based on clinical criteria (N = 2217). Legend: Total N = 2217 adjudicated hospitalizations. Implicit/Explicit ICD-9 sepsis defined based on taxonomies of infection and organ dysfunction codes proposed by Angus, et al. in addition to explicit sepsis codes. CMS ICD-9 sepsis defined based on list of codes used in the CMS measure. REGARDS-EHR defined as infection event meeting modified EHR criteria proposed by Rhee, et al.^4^ REGARDS-SOFA defined as infection event with ≥2 SOFA points across all organ systems (respiratory, cardiovascular, renal, hematological, hepatic, and neurological). REGARDS-qSOFA defined as infection events meeting ≥2 qSOFA criteria. Panel A) Kappa statistics and observed agreement; Panel B) Sensitivity and specificity with clinical criteria as standards. Bias-corrected 95% confidence intervals obtained by bootstrapping and shown in parentheses for all measures. SOFA = sepsis-related organ failure assessment; qSOFA = “quick” sepsis-related organ failure assessment; EHR = electronic health record. CMS = Centers for Medicare and Medicaid Services; ICD-9 = International Classification of Diseases, Ninth Revision

Overall agreement between the Medicare-Implicit/Explicit and REGARDS-SOFA, REGARDS-qSOFA, and REGARDS-EHR was 77, 79, 81%, respectively, and between Medicare-CMS and REGARDS-SOFA, REGARDS-qSOFA, and REGARDS-EHR was 77, 87, 85%, respectively (Fig. 2a-f). The Kappa statistics for Medicare-Implicit/Explicit and Medicare-CMS were below 0.4 for all comparisons, but highest for REGARDS-EHR as compared with REGARDS-SOFA and REGARDS-qSOFA (Fig. 3a). Across all comparisons, the sensitivity estimates for claims-based approaches were below 60%, with lower values for Medicare-CMS as compared with Medicare-Implicit/Explicit (Fig. 3b). In contrast, the specificity was above 80% for all comparisons and was higher for Medicare-CMS compared with Medicare-Implicit/Explicit. PPVs were below 60% and were higher for Medicare-CMS as compared with Medicare-Implicit/Explicit (Additional file 2: Appendix G of the Online Results Supplement). NPVs for Medicare-Implicit/Explicit and Medicare-CMS were similar and 80% or above for all comparisons. Sensitivity of Medicare-Implicit/Explicit and Medicare-CMS was higher when we restricted the analysis to events occurring in 2009–2012 as compared with the main analysis (Additional file 2: Appendix H of the Online Results Supplement).

Overall, 1327 (59.9%) of the 2217 suspected infection events detected through REGARDS study procedures had infection as the primary reason for hospitalization and were identified through episodes of care defined using Medicare claims (Additional file 2: Appendix I of the Online Results Supplement). As compared with the main analysis, sensitivity estimates for both claims-based approaches were higher, and specificity was lower when restricted to events in this subgroup (Additional file 2: Appendix J of the Online Results Supplement).

### Comparison of mortality across concordant and discordant groups

**Fig. 4:**
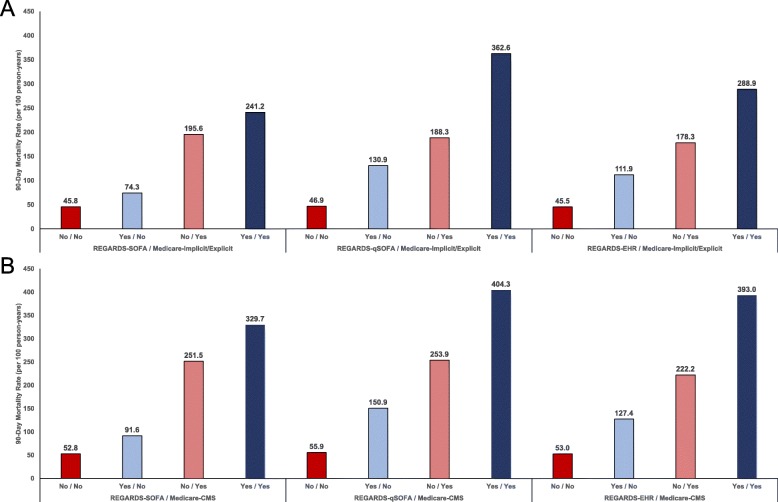
90-Day mortality rates by claims-based methods for sepsis identification and abstracted clinical criteria (N = 2217). Legend: Total N = 2217 adjudicated hospitalizations. Implicit/Explicit ICD-9 sepsis defined based on taxonomies of infection and organ dysfunction codes proposed by Angus, et al. in addition to explicit sepsis codes. CMS ICD-9 sepsis defined based on list of codes used in the CMS measure. REGARDS-EHR defined as infection event meeting modified EHR criteria proposed by Rhee, et al.^4^ REGARDS-SOFA defined as infection event with ≥2 SOFA points across all organ systems (respiratory, cardiovascular, renal, hematological, hepatic, and neurological). REGARDS-qSOFA defined as infection events meeting ≥2 qSOFA criteria. Rates include in-hospital deaths. Panel A) Comparisons of REGARDS clinical criteria with Medicare-Implicit/Explicit; Panel B) Comparisons of REGARDS clinical criteria with Medicare-CMS. SOFA = sepsis-related organ failure assessment; qSOFA = “quick” sepsis-related organ failure assessment; EHR = electronic health record. CMS = Centers for Medicare and Medicaid Services; ICD-9 = International Classification of Diseases, Ninth Revision; PY = person-years

Events meeting the REGARDS-SOFA classification had a lower overall 90-day mortality rate (140.7 per 100 person-years) compared with the Medicare-CMS (296.1 per 100 person-years), REGARDS-qSOFA (238.6 per 100 person-years), Medicare-Implicit/Explicit (219.4 per 100 person-years), and REGARDS-EHR classifications (201.8 per 100 person-years). Figure 4a and b show the 90-day mortality rate per 100 person-years within concordant and discordant groups defined by the cross-tabulation of each claims-based algorithm with each clinical definition of sepsis. Across all concordant and discordant groups, events meeting claims-based sepsis definitions (i.e., Medicare-Implicit/Explicit and Medicare-CMS) had higher 90-day mortality versus those not meeting claims-based definitions. For events not meeting claims-based sepsis definitions, those meeting versus not meeting clinical criteria for sepsis had higher 90-day mortality. For events meeting claims-based sepsis definitions, those meeting versus not meeting classifications based on clinical criteria also had higher 90-day mortality. Meeting versus not meeting claims-based sepsis definitions was associated with a higher mortality rate after multivariable adjustment (Additional file 2: Appendix K of the Online Results Supplement). After multivariable adjustment, REGARDS-qSOFA and REGARDS-EHR, but not REGARDS-SOFA, were associated with higher mortality for events not meeting claims-based definitions.

## Discussion

In the current study, claims-based algorithms had high observed agreement and specificity but low sensitivity when compared with sepsis classifications based on updated clinical criteria. In addition, across all clinical criteria, PPVs for claims-based algorithms were low and NPVs were high. Also, 90-day mortality rates were similar between the claims-based methods, qSOFA criteria, and EHR criteria but these approaches identified a population with higher mortality relative to sepsis as defined using SOFA.

The current study builds on prior work comparing sepsis diagnosis code algorithms with clinical criteria [4, 12–15, 30]. Rhee, et al., pooled data from over 400 hospitals for 2009–2014 and compared the validity of the modified Angus method (i.e., “implicit” or “explicit” codes for sepsis) using SOFA as the gold standard [4]. For the Angus method, sensitivity was substantially higher (66% versus 53%) while specificity was slightly higher (90% versus 85%) when compared with estimates from 2009 to 2012 in the current study. For this same period in the current study, the Angus method had higher sensitivity (60% versus 53%) and similar specificity (82% versus 85%) when we specified EHR criteria as the gold standard instead of SOFA. In addition, Iwashyna, et al. performed a patient-level validation of discharge diagnosis-based methods and the authors reported similar sensitivity (50%) and higher specificity (96%) using chart-reviewed infection with SIRS-related organ dysfunction as the standard [12]. Whittaker, et al. also examined the Angus method in a retrospective analysis of cases meeting SIRS criteria with organ dysfunction, finding similar sensitivity as compared with our study (47%) [13]. Thus, we demonstrate that despite high overall agreement, sensitivity is low for claims-based sepsis algorithms when using updated clinical criteria and adjudicated infection events from one of the largest cohort studies in the US.

In the REGARDS population, claims-based sepsis algorithms had high observed agreement with clinical criteria, but low sensitivity for identifying sepsis events. Based on our subgroup analysis using episodes of care in Medicare with infection as the primary reason for hospitalization, infection coding did not explain the low sensitivity of these algorithms. However, incomplete or inaccurate coding of explicit sepsis or organ dysfunction could lead to reduced sensitivity. Although the proportion of hospitalizations with coded organ dysfunction has increased over time, prior studies suggest that organ dysfunction may not be coded accurately [4, 30, 31]. Alternatively, patients may meet SOFA, qSOFA, or EHR criteria without having a diagnosis of organ dysfunction. It is also possible that the low sensitivity of claims-based algorithms for identifying sepsis could be explained by the lack of a true gold standard definition. Proposed clinical criteria have attempted to map organ dysfunction in specific systems to discrete physiological manifestations, but it is unclear whether these approaches should be used as standards for comparison with diagnosis code algorithms [6, 7, 19]. Given these limitations, it may be preferable to focus on comparisons of claims-based approaches with more objective methods for defining sepsis, such as the previously derived EHR definition also assessed in the current study [4].

A published framework describes predictive validity (i.e., the ability for a definition to identify infection patients with increased risk of mortality) as an important consideration for evaluating sepsis definitions [6]. In the current study, claims-based algorithms identified a population with higher 90-day mortality than SOFA criteria, but similar mortality to qSOFA and EHR criteria. This supports the use of claims-based algorithms to retrospectively identify patients with infection at high-risk of mortality when laboratory measures are not available. But, to ensure that a high proportion of true sepsis cases are identified, regulatory bodies should consider approaches with high sensitivity in the context of quality improvement initiatives tracking compliance with specific treatment guidelines. Although objective clinical criteria or approaches with higher sensitivity should be used whenever possible, further research is needed to determine the role of claims-based algorithms and the optimal definitions of sepsis in specific contexts (e.g., research in resource-limited settings, surveillance and prevention efforts, quality improvement, or clinical care).

Our study has several strengths, including use of data from a large contemporary population-based cohort and the availability of comprehensive clinical data. We assembled suspected infection events which were subsequently confirmed via chart abstraction as part of a prospective cohort study. This approach, combined with the sensitivity analysis restricting to events present in claims, minimizes the possibility that differences in infection detection or missing claims would explain why events may have met clinical criteria without having sepsis diagnoses. However, results from the current study should be interpreted considering several limitations. Although we identified a large sample of infection events, the method of detection in REGARDS cannot be considered true surveillance and we may have missed events during follow-up. Therefore, events detected in the current study may not generalize to all infection events. It is also possible that claims episodes were missed, as sources other than Medicare may have been billed for a given hospitalization. Miscoding of a claims-defined episode is possible because data on physician diagnosis codes were collected for billing purposes and not research. In addition, abstraction was limited to the first 28 h of a hospitalization and we could not identify “secondary” or “evolving” sepsis using this approach. However, this allows for greater confidence that infections and organ dysfunctions identified via chart abstraction were related. The current analysis was limited to black and white Medicare beneficiaries 65 years of age or older. Therefore, the current results also may not generalize to younger populations or other race/ethnic groups. However, compared to estimates obtained from a large-scale study of sepsis in the US, population characteristics and outcomes of infection events were similar [4].

## Conclusion

In conclusion, the Medicare-Implicit/Explicit and Medicare-CMS claims-based algorithms for identifying sepsis had high observed agreement with clinical criteria derived from updated guidelines but had low sensitivity. Among suspected infection hospitalizations, claims-based methods identified a population with higher risk of mortality as compared with SOFA criteria. In contexts requiring clinical granularity and high sensitivity, such as national quality improvement initiatives, classifications based on clinical criteria may be preferable over claims-based algorithms.

## Supplementary information

**Additional file 1: Appendix A.** Diagram of the process for identification and adjudication of chart-abstracted infection and clinical criteria in REGARDS. **Appendix B.** Infection Screening and Abstraction Taxonomy. **Appendix C.** Diagram of the process for identification of infection and sepsis episodes using claims-based ICD-9 code algorithms. **Appendix D.** Discharge diagnosis codes used in claims-based methods of sepsis identification. **Appendix E.** Detailed definitions and technical information for demographics, health-related factors, chronic medical conditions, and biomarkers.

**Additional file 2: Appendix F.** Percent of REGARDS adjudicated hospitalizations with primary infections meeting criteria over the study period, 2003–2012 (*N* = 1271). **Appendix G.** Positive and negative predictive values for claims-based methods among REGARDS adjudicated hospitalizations (*N* = 2217). **Appendix H.** Agreement and measures of validity for claims-based methods among REGARDS adjudicated hospitalizations for years 2009–2012 (*N* = 1044). **Appendix I.** Participant characteristics for suspected infection events with infection as a primary reason for hospitalization in the REGARDS study which were present in Medicare episodes of care versus other REGARDS suspected infection events (*N* = 2217). **Appendix J.** Agreement and measures of validity for claims-based methods among suspected infection events with infection as a primary reason for hospitalization in the REGARDS study which were present in Medicare episodes of care (*N* = 1054). **Appendix K.** Mortality rates and hazard ratios for 90-Day mortality by claims-based methods for sepsis identification and abstracted clinical criteria (*N* = 2217).

## Data Availability

This study uses data from the REGARDS cohort. In order to abide by its obligations with NIH/NINDS and the institutional review board of the University of Alabama at Birmingham, REGARDS facilitates data sharing through formal data use agreements. Any investigator is welcome to access the REGARDS data through this process. Requests for data access may be sent to regardsadmin@uab.edu.

## Abbreviations

CI: Confidence interval
CKD: Chronic kidney disease
CMS: Centers for Medicare and Medicaid Services
ED: Emergency Department
EHR: Electronic health record
ICD-10: International Classification of Diseases 10th Revision
ICD-9: International Classification of Diseases 9th Revision
MI: Myocardial infarction
NPV: Negative predictive value
PPV: Positive predictive value
qSOFA: “Quick” sepsis-related organ failure assessment
REGARDS: Reasons for Geographic and Racial Differences in Stroke
SIRS: Systemic inflammatory response syndrome
SOFA: Sepsis-related organ failure assessment
US: United States
