# Supplementary material for: Agreement of claims-based methods for identifying sepsis with clinical criteria in the REasons for Geographic and Racial Differences in Stroke (REGARDS) cohort
Source: BMC Med Res Methodol. 2020 Mar 4;20:54. doi: 10.1186/s12874-020-00937-9 (PMC7057471; doi:10.1186/s12874-020-00937-9)
Supplement: Supplementary file 1 — Additional file 1: Appendix A. Diagram of the process for identification and adjudication of chart-abstracted infection and clinical criteria in REGARDS. Appendix B. Infection Screening and Abstraction Taxonomy. Appendix C. Diagram of the process for identification of infection and sepsis episodes using claims-based ICD-9 code algorithms. Appendix D. Discharge diagnosis codes used in claims-based methods of sepsis identification. Appendix E. Detailed definitions and technical information for demographics, health-related factors, chronic medical conditions, and biomarkers. [file 12874_2020_937_MOESM1_ESM.pdf]

ONLINE METHODS SUPPLEMENT

APPENDIX A Diagram of the process for identification and adjudication of chart-abstracted infection and clinical criteria in REGARDS

APPENDIX B Infection Screening and Abstraction Taxonomy

APPENDIX C Diagram of the process for identification of infection and sepsis episodes using claims-based ICD-9 code algorithms

APPENDIX D Discharge diagnosis codes used in claims-based methods of sepsis identification

APPENDIX E Detailed definitions and technical information for demographics, health-related factors, chronic medical conditions, and biomarkers

APPENDIX A Diagram of the process for identification and adjudication of chart-abstracted infection and clinical criteria in REGARDS

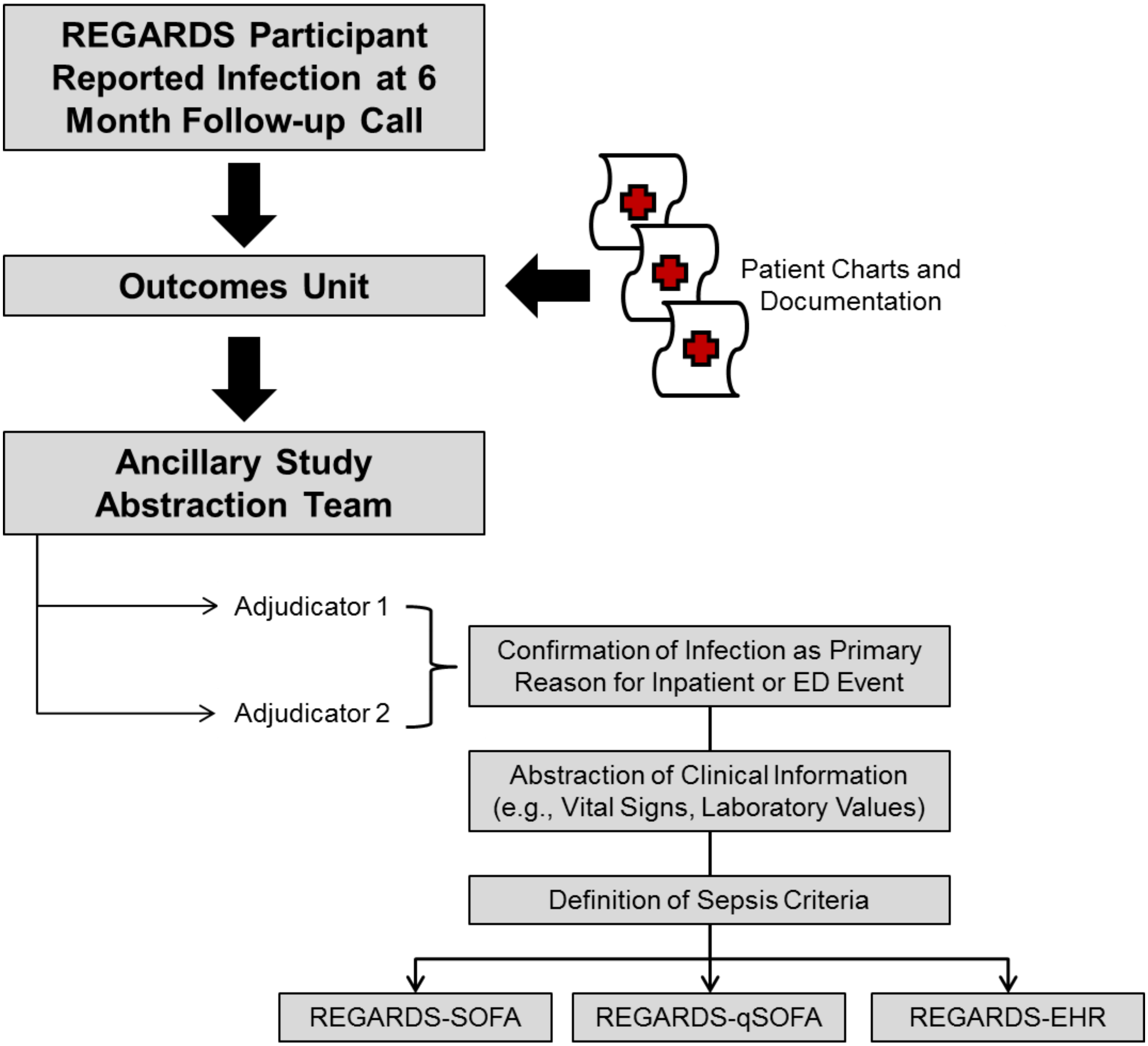

APPENDIX A Legend: REGARDS-EHR defined as infection event meeting modified EHR criteria proposed by Rhee, et al.[4] REGARDS-SOFA defined as infection event with ≥ 2 SOFA points across all organ systems (respiratory, cardiovascular, renal, hematological, hepatic, and neurological). REGARDS-qSOFA defined as infection events meeting ≥ 2 qSOFA criteria. ED = Emergency Department; REGARDS = Reasons for Geographic and Racial Differences in Stroke study; SOFA = sepsis-related organ failure assessment; qSOFA = “quick” sepsis-related organ failure assessment; EHR = electronic health record.

APPENDIX B

INFECTION SCREENING AND ABSTRACTION TAXONOMY

Hospitalizations will be classified as Infection – Maybe Infection – Not Infection:

- Infection – Hospitalization associated with significant microbial infestation and illness
- Maybe Infection – Lesser infections (cold, flu, upper respiratory) or unclear connection to significant disease

Use Angus, et al., CCM 2001 as guideline.

If “same ailment” listed, take categorization of prior admission.

If any keyword is an infection or maybe-infection, classify accordingly.

Use the following list of keywords to identify potential serious infections among the REGARDS “other hospitalizations” and “nurses review” lists (See Table).

Identify only events where serious infection was a primary reason for hospitalization:

- Include cases where infection was one of several primary reasons for hospitalization. For example, a patient admitted for heart failure and pneumonia.
- Include cases where a minor infection progressed to a serious infection. For example, viral syndrome progressed to pneumonia.
- Include “ER-only” visits. These cases occasionally have serious infection.
- Do NOT include cases where the infection was a secondary reason for hospitalization. For example, a patient admitted for heart failure, but then aspirated and developed pneumonia.
- Do NOT include “outpatient-only” visits.

INFECTION	MAYBE INFECTION<br>(Potential but Lower Likelihood of Sepsis)	NOT INFECTION
**LUNG INFECTIONS**<br>Bronchitis, Pneumonia	**BREATHING PROBLEMS**<br>Asthma, Breathing Problem, Fluid in Lung	Organ or Part without Further Info ("stomach")<br>Arthritis<br>Cyst on Kidney<br>Fungal Infection of Toe<br>Ganglion Cyst<br>Gastritis<br>Gout<br>Growth<br>Hiatal Hernia<br>Interstitial Cystitis<br>Kidney Stones<br>Ovarian Cyst<br>Pleurisy<br>Stomach Blockage<br>Testicular/Scrotal Cyst<br>Stomach Ulcer, Reflux, Endoscopy, Bleeding<br>Rash w/out Indication<br>Gall Stones, Cholecystectomy<br>Sweating, Hot, Heat Exhaustion
**KIDNEY INFECTIONS (GENITOURINARY)**<br>Bladder Infection<br>STDs / Pelvic Inflam Dis<br>Cystitis<br>Urinary Tract Infect<br>Prostatitis<br>Testicle Infection	**FLU, VIRUS, COLD**<br>Cold<br>Upper Respiratory Infection<br>Cough (Non-Specific)<br>Viral Syndrome<br>Flu<br>Virus<br>Respiratory Infection	
**SKIN AND EXTREMITY INFECTIONS**<br>Abscess/Boil<br>Infected Extremity<br>Cellulitis<br>Osteomyelitis<br>Cyst Removal<br>Phlebitis<br>Extremity Cyst<br>Shingles<br>Gangrene<br>Skin Cyst<br>Infected Cut<br>Vaginal Cyst	**EAR, NOSE, AND THROAT**<br>Ear Ache<br>Sinusitis<br>Ear Infection<br>Strep Throat<br>Mouth Infection<br>Throat Infection<br>Pharyngitis<br>Tonsillitis<br>Sinus Infection<br>Tooth Infection/Abscess	
**ABDOMINAL INFECTIONS**<br>Appendicitis/Appendectomy<br>Food Poisoning/Gastroenteritis<br>Cholecystitis<br>Hepatitis<br>Colitis/Crohn's<br>Intestinal Infection<br>Diarrhea<br>Ischemic Colitis<br>Diverticulitis<br>Liver Infection<br>Pancreatitis<br>Rectal Abscess<br>Ulcerative Colitis<br>Peritonitis	**ABDOMINAL**<br>Intestinal Virus<br>Stomach Problem<br>Stomach Infection<br>Stomach Virus<br>Stomach/ Abdominal Pain<br>Vomit/Nausea w/out Diarrhea (or other indication)	
**SYSTEMIC INFECTION/SEPSIS**<br>Blood Infection, Sepsis	**OTHER**<br>Antibiotic (Unspecified)<br>Fever w/ Cold/Flu<br>Conjunctivitis<br>Virus or Viral Infect<br>Dehydration<br>Yeast Infection<br>Eye Infection	
**OTHER**<br>Meningitis<br>Pericarditis<br>Bone Infection<br>Staph Infection<br>Chills or Fever Alone<br>White Blood Cells – Low/High<br>Infection (Unspecified)<br>Malaria		

APPENDIX C Diagram of the process for identification of infection and sepsis episodes using claims-based ICD-9 code algorithms

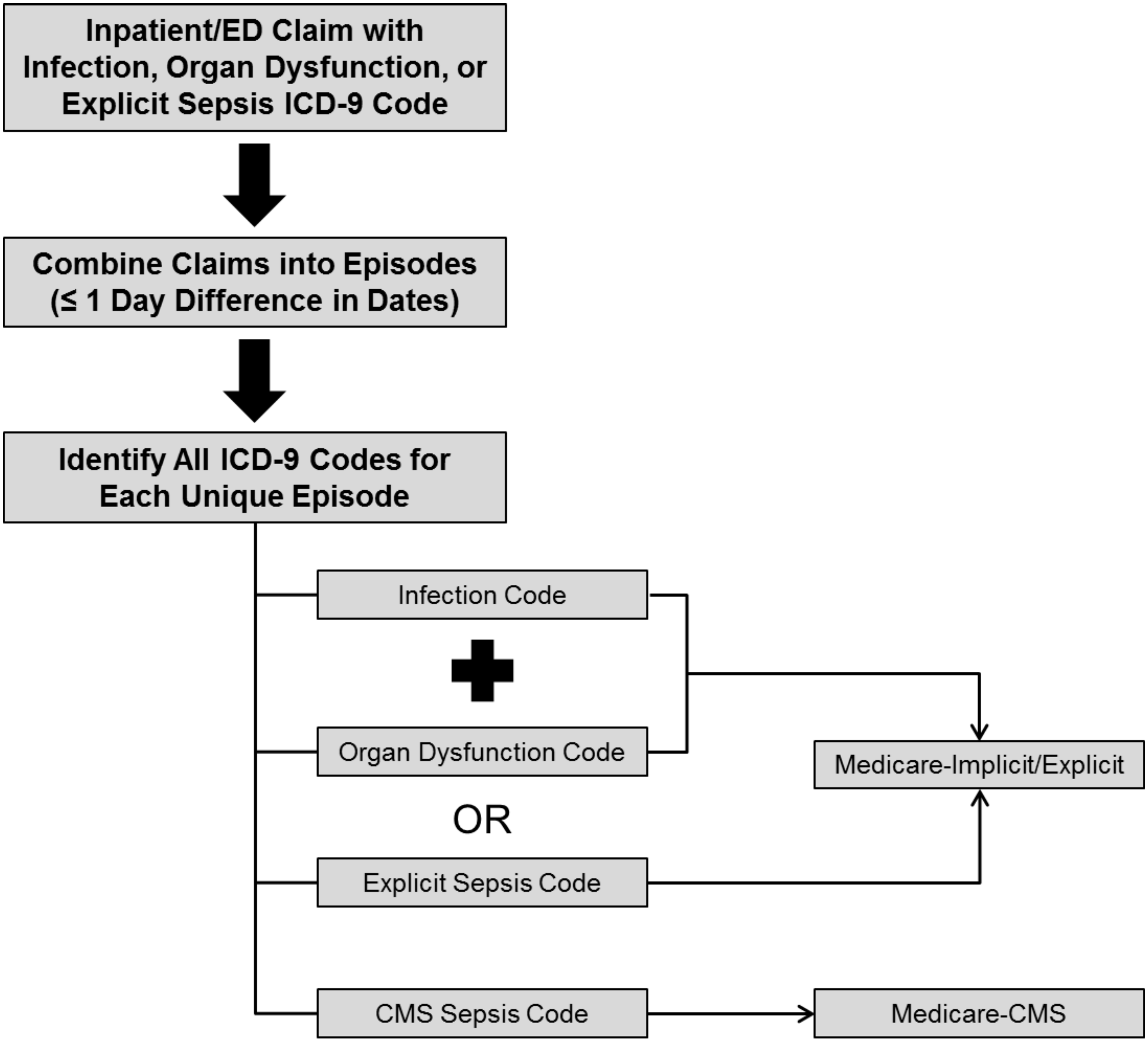

APPENDIX C Legend: Implicit/Explicit ICD-9 sepsis defined based on taxonomies of infection and organ dysfunction codes proposed by Angus, et al. in addition to explicit sepsis codes. CMS ICD-9 sepsis defined based on list of codes used in the CMS measure. ED = Emergency Department; CMS = Centers for Medicare and Medicaid Services; ICD-9 = International Classification of Diseases, Ninth Revision.

APPENDIX D Discharge diagnosis codes used in claims-based methods of sepsis identification

Claims-based Method	ICD-9-CM Codes
**Medicare-Implicit/Explicit (1)**	
**Infection**	
Infectious and Parasitic Diseases	001-005, 008-008.5, 009, 013, 018, 020-027, 032-041, 098, 100-101, 112, 112.4, 112.5, 112.8, 114-118
Neurologic	320, 321, 321.1, 324, 325, 360, 376, 380.14, 383
Circulatory	420.99, 421
Respiratory	461-465, 475, 481-482,<br>485-486, 491.21, 494, 510, 513
Digestive	522.5, 522.7, 526.4, 527.3, 528.3, 540-542, 562.01, 562.03, 562.11, 562.13, 566-567, 569.5, 569.61, 569.83, 572, 572.1, 575
Genitourinary	590, 599, 601, 604, 614, 615, 616.3, 616.4
Pregnancy	634-639, 646.6, 658.4, 670, 675.1
Skin	681-683, 685-686
Musculoskeletal	711, 728.86, 730
Other	790.7, 958.3, 996.6, 998.5, 999.3
Sepsis	995.91
**Organ Dysfunction**	
Cardiovascular	458, 458.8, 458.9, 785.5
Hematologic	286.6, 286.9, 287.4, 287.5
Hepatic	570, 573.4
Neurologic	293, 348.1, 348.3
Renal	584
Respiratory	518.8, 786.03, 799.1
**Explicitly-Coded Severe Sepsis**	
Severe Sepsis	995.92
Septic Shock	785.52
**Medicare-CMS (ICD-10→ICD-9) (2)**	A02.1→ 003.1, A22.7→ 022.3, A26.7→ 995.91, A32.7→ 995.91, A40.0→ 038.0, A40.1→ 038.0, A40.3→ 038.2, A40.8→ 038.0, A40.9→ 038.0, A41.01→ 038.11, A41.02→ 038.12, A41.1→ 038.19, A41.2→ 038.10, A41.3→ 038.41, A41.4→ 038.3, A41.50→ 038.40, A41.51→ 038.42, A41.52→ 038.43, A41.53→ 038.44, A41.59→ 038.49, A41.81→ 038.8, A41.89→ 038.8, A41.9→ 038.9, A42.7→ 038.8, A54.86→ 995.91, B37.7→ 112.5, R65.20→ 995.92, R65.21→ 785.52

APPENDIX D Legend: ICD-9 = International Classification of Diseases, Ninth Revision; ICD-10 = International Classification of Diseases, Tenth Revision; CMS = Centers for Medicare and Medicaid Services.

Relevant Citations:

1) Angus DC, Linde-Zwirble WT, Lidicker J, Clermont G, Carcillo J, Pinsky MR. Epidemiology of severe sepsis in the United States: analysis of incidence, outcome, and associated costs of care. Critical care medicine. 2001;29(7):1303-1310.

2) Specifications Manual, Version 5.2a. QualityNet https://www.qualitynet.org/dcs/ContentServer?c=Page&pagename=QnetPublic%2FPage%2FQnetTier4&cid=1228775749207. Accessed May 13th, 2017.

APPENDIX E Detailed definitions and technical information for demographics, health-related factors, chronic medical conditions, and biomarkers

Characteristic	Definition and/or Technical Information
**Demographics**	
Age	Age in years at the time of hospitalization
Gender	Male, Female
Race	Black/African American, White
Education	Participant reported:<br>- Less than high school<br>- High school graduate<br>- Some college<br>- College or higher<br>- Missing
Income	Participant reported:<br>- <$20k<br>- $20k-$34k<br>- $35k-$74k<br>- ≥$75k<br>- Missing (not reported)
**Health Behaviors**	
Smoking Status	Participant reported:<br>- Current<br>- Past<br>- Never
Alcohol Use	Participant reported (1):<br>- Heavy (>1 drink per day for women and >2 drinks per day for men)<br>- Moderate (≤1 drink per day for women or ≤2 drinks per day for men)<br>- None
**Medical Conditions**	
Atrial Fibrillation	Participant reported history of atrial fibrillation or baseline electrocardiographic evidence of atrial fibrillation.
Chronic Kidney Disease (CKD)	CKD defined as eGFR <60 mL/min/1.73m^2.<br>Serum creatinine was measured by colorimetric reflectance spectrophotometry (Ortho Vitros Clinical Chemistry System 950IRC, Johnson & Johnson Clinical Diagnostics, Raritan, New Jersey, USA). eGFR was calculated using the CKD-Epi equation. (3)
Diabetes	Fasting glucose ≥126 mg/L (or a glucose ≥200 mg/L for those not fasting) or participant reported use of insulin or oral hypoglycemic agents.
Dyslipidemia	Low-density lipoprotein cholesterol >130 mg/dL or participant reported use of lipid lowering medications.
Hypertension	Systolic blood pressure ≥140 mm Hg, diastolic blood pressure ≥90 mm Hg, or participant reported antihypertensive agent use.
Lung Disease	Participant use of pulmonary medications (beta agonists, leukotriene inhibitors, inhaled corticosteroids, combination inhalers, ipratropium, cromolyn, aminophylline and theophylline).
Myocardial Infarction	Participant reported history of myocardial infarction or baseline electrocardiographic evidence of myocardial infarction.
Obesity	Defined based on BMI [>30 kg/m^2] or waist circumference [>102 cm for males or >88 cm for females] using measurements from the in home visit. (2)
Stroke	Participant reported history of stroke or transient ischemic attack.

APPENDIX E Legend: BMI = body mass index; eGFR = estimated glomerular filtration rate; CKD = chronic kidney disease.

Relevant Citations:

(1) National Institute on Alcohol Abuse and Alcoholism. Helping Patients Who Drink Too Much, a Clinician's Guide. 2005 [cited 2012 February 13]Available from: http://pubs.niaaa.nih.gov/publications/Practitioner/CliniciansGuide2005/guide.pdf

(2) Janssen I, Katzmarzyk PT, Ross R. Body mass index, waist circumference, and health risk: evidence in support of current National Institutes of Health guidelines. Arch Intern Med. Oct 14 2002;162(18):2074-2079.

(3) James MT, Hemmelgarn BR, Wiebe N, et al. Glomerular filtration rate, proteinuria, and the incidence and consequences of acute kidney injury: a cohort study. Lancet. Dec 18 2010;376(9758):2096-2103.
