# Supplementary material for: Agreement of claims-based methods for identifying sepsis with clinical criteria in the REasons for Geographic and Racial Differences in Stroke (REGARDS) cohort
Source: BMC Med Res Methodol. 2020 Mar 4;20:54. doi: 10.1186/s12874-020-00937-9 (PMC7057471; doi:10.1186/s12874-020-00937-9)
Supplement: Supplementary file 2 — Additional file 2: Appendix F. Percent of REGARDS adjudicated hospitalizations with primary infections meeting criteria over the study period, 2003–2012 (N = 1271). Appendix G. Positive and negative predictive values for claims-based methods among REGARDS adjudicated hospitalizations (N = 2217). Appendix H. Agreement and measures of validity for claims-based methods among REGARDS adjudicated hospitalizations for years 2009–2012 (N = 1044). Appendix I. Participant characteristics for suspected infection events with infection as a primary reason for hospitalization in the REGARDS study which were present in Medicare episodes of care versus other REGARDS suspected infection events (N = 2217). Appendix J. Agreement and measures of validity for claims-based methods among suspected infection events with infection as a primary reason for hospitalization in the REGARDS study which were present in Medicare episodes of care (N = 1054). Appendix K. Mortality rates and hazard ratios for 90-Day mortality by claims-based methods for sepsis identification and abstracted clinical criteria (N = 2217). [file 12874_2020_937_MOESM2_ESM.pdf]

# ONLINE RESULTS SUPPLEMENT

**APPENDIX F** Percent of REGARDS adjudicated hospitalizations with primary infections meeting criteria over the study period, 2003-2012 (N=1,271)

**APPENDIX G** Positive and negative predictive values for claims-based methods among REGARDS adjudicated hospitalizations (N=2,217)

**APPENDIX H** Agreement and measures of validity for claims-based methods among REGARDS adjudicated hospitalizations for years 2009-2012 (N=1,044)

**APPENDIX I** Participant characteristics for suspected infection events with infection as a primary reason for hospitalization in the REGARDS study which were present in Medicare episodes of care versus other REGARDS suspected infection events (N=2,217)

**APPENDIX J** Agreement and measures of validity for claims-based methods among suspected infection events with infection as a primary reason for hospitalization in the REGARDS study which were present in Medicare episodes of care (N=1,054)

**APPENDIX K** Mortality rates and hazard ratios for 90-Day mortality by claims-based methods for sepsis identification and abstracted clinical criteria (N=2,217)

**APPENDIX F** Percent of REGARDS adjudicated hospitalizations with primary infections meeting criteria over the study period, 2003-2012 (N=1,271)

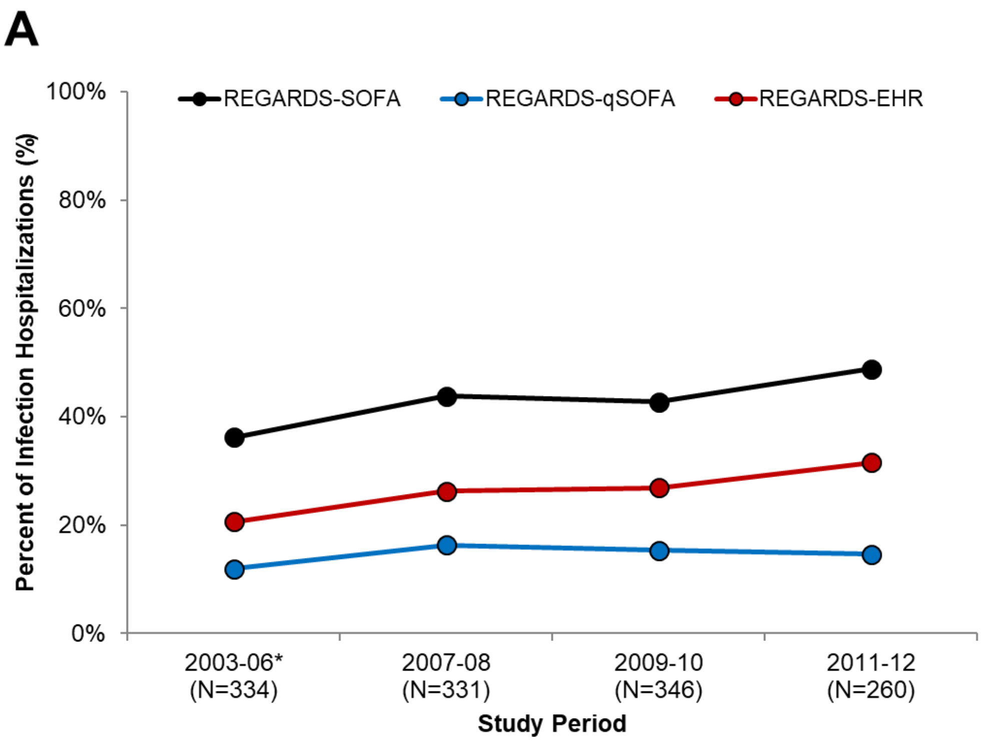


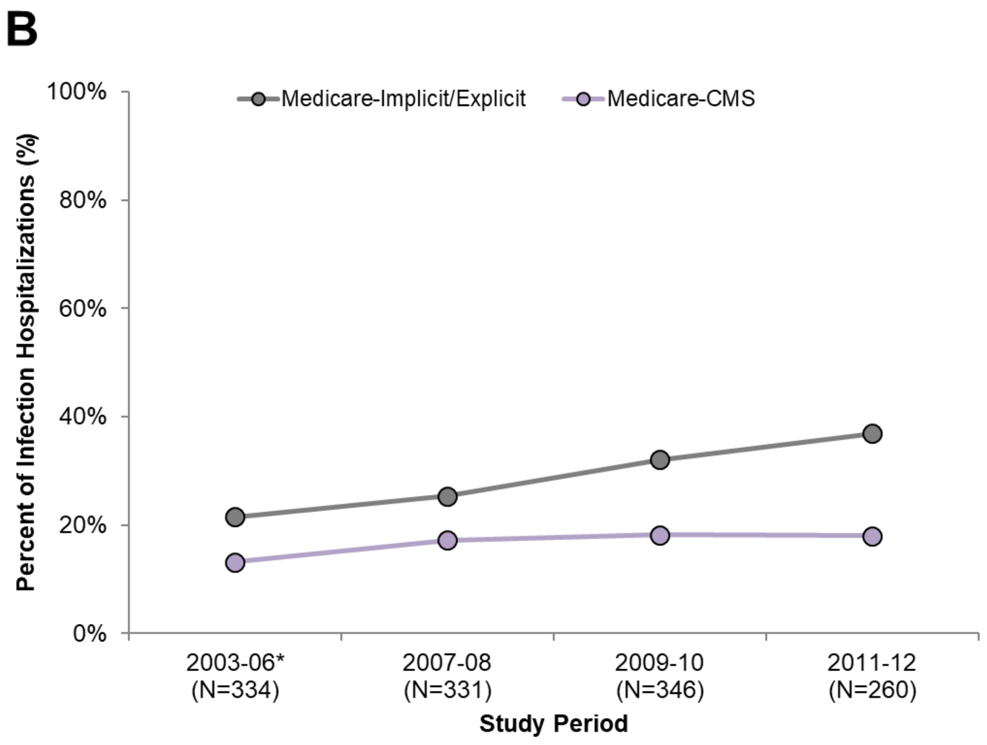

**APPENDIX F Legend:** Total N = 1,271 adjudicated hospitalizations with infection as a primary reason for hospitalization. Angus ICD-9 sepsis defined based on taxonomies of infection and organ dysfunction codes. CMS ICD-9 sepsis defined based on list of codes used in the CMS SEP-1 measure. REGARDS-SOFA defined as infection event with ≥ 2 SOFA points. REGARDS-qSOFA defined as infection event meeting ≥ 2 qSOFA criteria. Panel A) Trends for abstracted clinical criteria; Panel B) Trends for claims-based approaches. *Years combined due to small cell sizes. SOFA = sepsis-related organ failure assessment; qSOFA = “quick” sepsis-related organ failure assessment; CMS = Centers for Medicare and Medicaid Services; ICD-9 = International Classification of Diseases, Ninth Revision.

**APPENDIX G** Positive and negative predictive values for claims-based methods among REGARDS adjudicated hospitalizations (N=2,217)

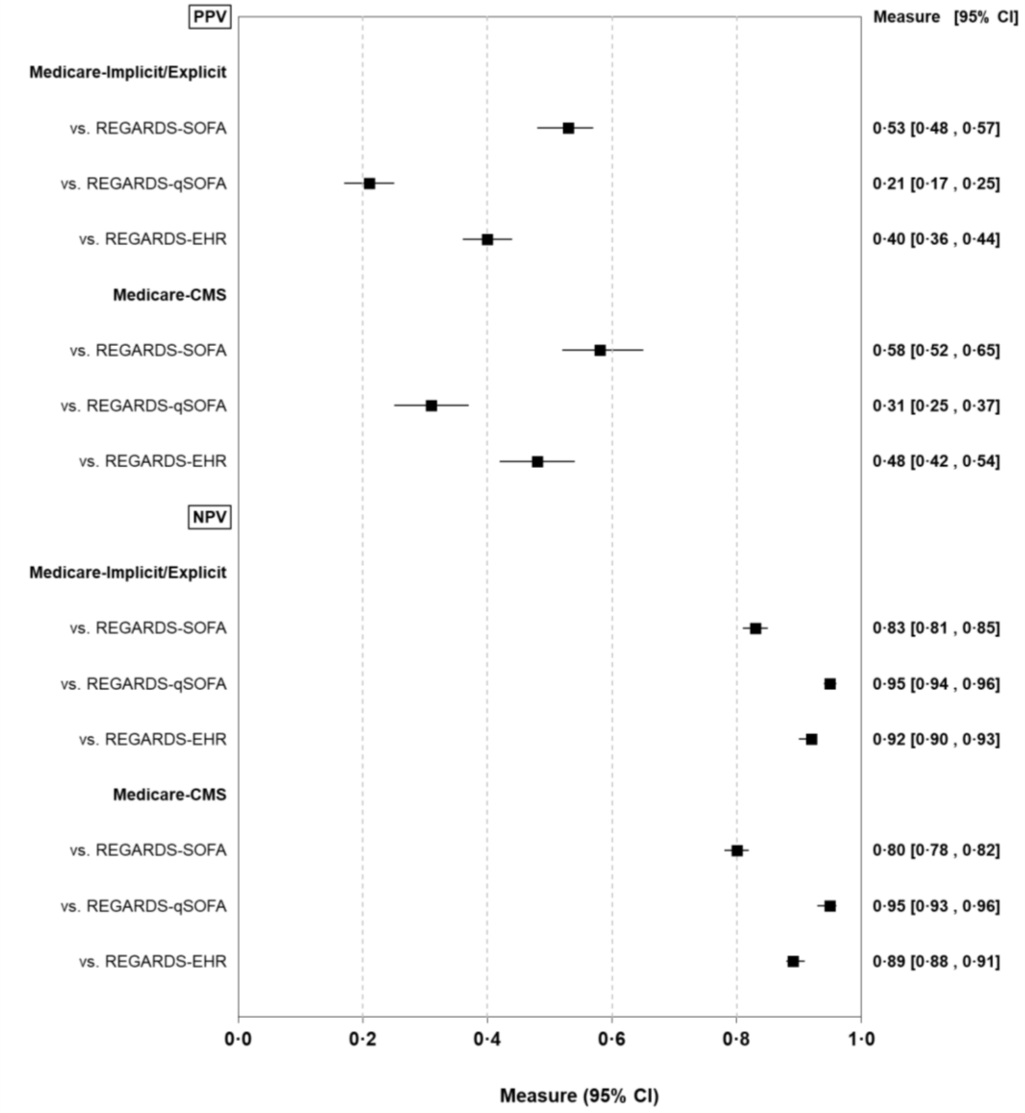

**APPENDI G Legend:** Total N = 2,217 adjudicated hospitalizations. Implicit/Explicit ICD-9 sepsis defined based on taxonomies of infection and organ dysfunction codes proposed by Angus, et al. in addition to explicit sepsis codes. CMS ICD-9 sepsis defined based on list of codes used in the CMS measure. REGARDS-EHR defined as infection event meeting modified EHR criteria proposed by Rhee, et al.[4] REGARDS-SOFA defined as infection event with ≥ 2 SOFA points across all organ systems (respiratory, cardiovascular, renal, hematological, hepatic, and neurological). REGARDS-qSOFA defined as infection events meeting ≥ 2 qSOFA criteria. PPV and NPV calculated with clinical criteria as standards. Bias-corrected 95% confidence intervals obtained by bootstrapping and shown in parentheses for all measures. SOFA = sepsis-related organ failure assessment; qSOFA = “quick” sepsis-related organ failure assessment; EHR = electronic health record. CMS = Centers for Medicare and Medicaid Services; ICD-9 = International Classification of Diseases, Ninth Revision; PPV = positive predictive value; NPV = negative predictive value.

**APPENDIX H** Agreement and measures of validity for claims-based methods among REGARDS adjudicated hospitalizations for years 2009-2012 (N=1,044)

| **Comparison** | **Observed Agreement Kappa (95% CI)** | **Sensitivity Specificity (95% CI)** | **PPV NPV (95% CI)** |
|---|---|---|---|
| **Medicare-Implicit/Explicit vs REGARDS** | | | |
| REGARDS-SOFA | 76.7% (74.2-79.5)<br>0.39 (0.33-0.45) | 52.7% (46.8-58.9)<br>85.3% (82.6-87.8) | 56.2% (49.8-61.9)<br>83.5% (80.5-86.4) |
| REGARDS-qSOFA | 76.9% (74.2-79.5)<br>0.21 (0.14-0.27) | 59.3% (48.4-68.7)<br>78.6% (75.9-81.2) | 20.9% (15.6-26.3)<br>95.3% (93.7-96.7) |
| REGARDS-EHR | 78.6% (76.1-81.3)<br>0.36 (0.29-0.42) | 60.0% (53.0-67.9)<br>82.4% (79.8-85.1) | 40.7% (34.9-47.1)<br>91.1% (89.0-93.3) |
| **Medicare-CMS vs REGARDS** | | | |
| REGARDS-SOFA | 77.9% (75.3-80.8)<br>0.31 (0.24-0.38) | 30.9% (25.2-36.6)<br>94.7% (93.0-96.2) | 67.5% (58.6-0.75)<br>79.3% (76.6-82.3) |
| REGARDS-qSOFA | 87.3% (85.2-89.3)<br>0.32 (0.22-0.40) | 46.2% (34.1-56.0)<br>91.2% (89.3-92.9) | 33.3% (24.3-42.4)<br>94.7% (93.1-96.0) |
| REGARDS-EHR | 84.0% (81.6-86.3)<br>0.35 (0.27-0.43) | 38.3% (30.5-45.4)<br>93.2% (91.5-94.8) | 53.2% (43.9-61.5)<br>88.2% (86.0-90.4) |

**APPENDIX H Legend:** Total N = 1,044 adjudicated hospitalizations over 2009-2012. Implicit/Explicit ICD-9 sepsis defined based on taxonomies of infection and organ dysfunction codes proposed by Angus, et al. in addition to explicit sepsis codes. CMS ICD-9 sepsis defined based on list of codes used in the CMS measure. REGARDS-EHR defined as infection event meeting modified EHR criteria proposed by Rhee, et al.[4] REGARDS-SOFA defined as infection event with ≥ 2 SOFA points across all organ systems (respiratory, cardiovascular, renal, hematological, hepatic, and neurological). REGARDS-qSOFA defined as infection events meeting ≥ 2 qSOFA criteria. Bias-corrected 95% confidence intervals obtained by bootstrapping and shown in parentheses for all measures. SOFA = sepsis-related organ failure assessment; qSOFA = "quick" sepsis-related organ failure assessment; EHR = electronic health record. CMS = Centers for Medicare and Medicaid Services; ICD-9 = International Classification of Diseases, Ninth Revision; PPV = positive predictive value; NPV = negative predictive value; CI = confidence interval.

**APPENDIX I** Participant characteristics for suspected infection events with infection as a primary reason for hospitalization in the REGARDS study which were present in Medicare episodes of care versus other REGARDS suspected infection events (N=2,217)

| Characteristic | Not Matched N = 890 | Matched to Infection Episode in Claims N = 1,327 | |
|---|---|---|---|
| | | No Primary Infection N = 273 | Primary Infection N = 1,054 |
| | % | % | % |
| **Age (years) (Mean/SD)** | 77.0 (6.6) | 77.4 (6.3) | 77.3 (6.6) |
| **Gender** | | | |
| Male | 52.4 | 48.7 | 56.2 |
| Female | 47.6 | 51.3 | 43.8 |
| **Race** | | | |
| White | 77.0 | 72.9 | 80.1 |
| Black | 23.0 | 27.1 | 19.9 |
| **Education*** | | | |
| <High School | 12.9 | 16.5 | 15.4 |
| High School Graduate or Higher | 87.1 | 83.5 | 84.6 |
| **Income** | | | |
| <$20k | 18.8 | 22.3 | 21.6 |
| ≥$20k | 68.4 | 65.2 | 66.0 |
| Not Available | 12.8 | 12.5 | 12.3 |
| **Smoking**** | | | |
| Current | 10.3 | 12.5 | 8.8 |
| Past | 48.9 | 55.9 | 53.2 |
| Never | 40.9 | 31.6 | 38.0 |
| **Alcohol Use†** | | | |
| Heavy | 3.3 | N<11 (Supp) | 3.6 |
| Moderate | 30.9 | 33.0 | 26.5 |
| None | 65.8 | Supp | 69.9 |
| **Comorbidities** | | | |
| Atrial Fibrillation | 15.1 | 18.3 | 15.9 |
| Lung Disease | 16.5 | 28.6 | 20.0 |
| Chronic Kidney Disease‡ | 24.4 | 27.1 | 31.3 |
| Stroke | 11.7 | 12.5 | 11.9 |
| Myocardial Infarction | 20.7 | 23.8 | 25.0 |
| Hypertension | 62.9 | 65.9 | 67.7 |
| Dyslipidemia | 62.7 | 60.8 | 66.8 |
| Diabetes | 25.1 | 25.6 | 29.8 |
| Obesity | 49.9 | 53.1 | 56.0 |
| **Sepsis Classification** | | | |
| REGARDS-SOFA | 8.3 | 0 | 44.3 |
| REGARDS-qSOFA | 2.4 | 0 | 15.6 |
| REGARDS-EHR | 4.4 | 0 | 27.7 |

**APPENDIX I Legend:** Total N = 2,217 adjudicated hospitalizations *1 missing. **6 missing. †38 missing. ‡Chronic kidney disease defined using creatinine values and the CKD-EPI equation. N<11(Supp) indicates a cell size less than 11, which is suppressed per our data use agreement. SOFA = sepsis-related organ failure assessment; qSOFA = “quick” sepsis-related organ failure assessment; EHR = electronic health record; SD = standard deviation.

**APPENDIX J** Agreement and measures of validity for claims-based methods among suspected infection events with infection as a primary reason for hospitalization in the REGARDS study which were present in Medicare episodes of care (N=1,054)

| Comparison | Observed Agreement<br>Kappa<br>(95% CI) | Sensitivity<br>Specificity<br>(95% CI) | PPV<br>NPV<br>(95% CI) |
|---|---|---|---|
| **Medicare-Implicit/Explicit vs REGARDS** | | | |
| REGARDS-SOFA | 67.9% (64.8-70.8)<br>0.34 (0.28-0.39) | 52.7% (48.5-57.7)<br>80.1% (76.9-83.2) | 67.8% (63.1-72.8)<br>68.0% (64.1-72.0) |
| REGARDS-qSOFA | 68.6% (66.0-71.3)<br>0.20 (0.14-0.26) | 59.8% (52.2-67.5)<br>70.2% (67.2-73.4) | 27.0% (22.5-31.9)<br>90.4% (88.3-92.7) |
| REGARDS-EHR | 73.3% (70.5-76.1)<br>0.38 (0.32-0.44) | 64.0% (58.1-70.1)<br>76.9% (74.0-79.9) | 51.5% (46.2-57.0)<br>84.8% (81.8-87.8) |
| **Medicare-CMS vs REGARDS** | | | |
| REGARDS-SOFA | 63.2% (60.1-66.5)<br>0.21 (0.15-0.27) | 31.0% (26.4-35.7)<br>88.8% (86.0-91.4) | 68.7% (62.0-75.6)<br>61.8% (58.3-65.6) |
| REGARDS-qSOFA | 78.8% (76.3-81.3)<br>0.28 (0.21-0.35) | 46.3% (38.3-54.4)<br>84.8% (82.6-87.4) | 36.0% (29.1-43.0)<br>89.6% (87.3-91.5) |
| REGARDS-EHR | 74.9% (72.3-77.6)<br>0.31 (0.25-0.38) | 40.8% (35.0-46.9)<br>87.9% (85.5-90.3) | 56.4% (48.8-63.9)<br>79.5% (76.8-82.6) |

**APPENDIX J Legend:** Total N = 1,054 adjudicated events with infection as a primary reason for hospitalization matched to infection episodes in claims. Implicit/Explicit ICD-9 sepsis defined based on taxonomies of infection and organ dysfunction codes proposed by Angus, et al. in addition to explicit sepsis codes. CMS ICD-9 sepsis defined based on list of codes used in the CMS measure. REGARDS-EHR defined as infection event meeting modified EHR criteria proposed by Rhee, et al.[4] REGARDS-SOFA defined as infection event with ≥ 2 SOFA points across all organ systems (respiratory, cardiovascular, renal, hematological, hepatic, and neurological). REGARDS-qSOFA defined as infection events meeting ≥ 2 qSOFA criteria. Bias-corrected 95% confidence intervals obtained by bootstrapping and shown in parentheses for all measures. SOFA = sepsis-related organ failure assessment; qSOFA = “quick” sepsis-related organ failure assessment; EHR = electronic health record. CMS = Centers for Medicare and Medicaid Services; ICD-9 = International Classification of Diseases, Ninth Revision; PPV = positive predictive value; NPV = negative predictive value; CI = confidence interval.

**APPENDIX K** Hazard ratios for 90-Day mortality by claims-based methods for sepsis identification and abstracted clinical criteria (N=2,217)

| Identification Method Grouping | 90-Day Mortality (From Event Date)* | | | |
|---|---|---|---|---|
| | N Deaths (%) | Mortality Rate per 100 py | Crude** Hazard Ratio (95% CI) | Adjusted**† Hazard Ratio (95% CI) |
| **Medicare-Implicit/Explicit vs REGARDS** | | | | |
| **REGARDS-SOFA** | | | | |
| SOFA No / Implicit-Explicit No (N=1,454) | 151 (10.4) | 45.8 | Ref | Ref |
| SOFA Yes / Implicit-Explicit No (N=295) | 48 (16.3) | 74.3 | 1.59 (1.15-2.19) | 1.18 (0.83-1.67) |
| SOFA No / Implicit-Explicit Yes (N=222) | 77 (34.7) | 195.6 | 3.83 (2.89-5.07) | 3.30 (2.45-4.45) |
| SOFA Yes / Implicit-Explicit Yes (N=246) | 103 (41.9) | 241.2 | 4.60 (3.60-5.89) | 3.29 (2.47-4.37) |
| **REGARDS-qSOFA** | | | | |
| qSOFA No / Implicit-Explicit No (N=1,662) | 177 (10.6) | 46.9 | Ref | Ref |
| qSOFA Yes / Implicit-Explicit No (N=87) | 22 (25.3) | 130.9 | 2.64 (1.70-4.09) | 2.05 (1.31-3.21) |
| qSOFA No / Implicit-Explicit Yes (N=370) | 127 (34.3) | 188.3 | 3.63 (2.87-4.59) | 2.94 (2.28-3.80) |
| qSOFA Yes / Implicit-Explicit Yes (N=98) | 53 (54.1) | 362.6 | 6.27 (4.76-8.27) | 5.04 (3.69-6.89) |
| **REGARDS-EHR** | | | | |
| EHR No / Implicit-Explicit No (N=1,605) | 166 (10.3) | 45.5 | Ref | Ref |
| EHR Yes / Implicit-Explicit No (N=144) | 33 (22.9) | 111.9 | 2.35 (1.61-3.42) | 1.85 (1.22-2.80) |
| EHR No / Implicit-Explicit Yes (N=281) | 92 (32.7) | 178.3 | 3.55 (2.74-4.61) | 3.02 (2.29-3.99) |
| EHR Yes / Implicit-Explicit Yes (N=187) | 88 (47.1) | 288.9 | 5.40 (4.19-6.95) | 4.14 (3.07-5.59) |
| **Medicare-CMS vs REGARDS** | | | | |
| **REGARDS-SOFA** | | | | |
| SOFA No / CMS No (N=1,572) | 186 (11.8) | 52.8 | Ref | Ref |
| SOFA Yes / CMS No (N=396) | 78 (19.7) | 91.6 | 1.69 (1.30-2.19) | 1.24 (0.92-1.66) |
| SOFA No / CMS Yes (N=104) | 42 (40.4) | 251.5 | 4.21 (3.00-5.91) | 3.57 (2.46-5.18) |
| SOFA Yes / CMS Yes (N=145) | 73 (50.3) | 329.7 | 5.16 (3.96-6.72) | 3.77 (2.74-5.17) |
| **REGARDS-qSOFA** | | | | |
| qSOFA No / CMS No (N=1,859) | 233 (12.5) | 55.9 | Ref | Ref |
| qSOFA Yes / CMS No (N=109) | 31 (28.4) | 150.9 | 2.53 (1.77-3.62) | 2.03 (1.39-2.96) |
| qSOFA No / CMS Yes (N=173) | 71 (41.0) | 253.9 | 3.97 (3.04-5.19) | 3.24 (2.40-4.38) |
| qSOFA Yes / CMS Yes (N=76) | 44 (57.9) | 404.3 | 5.78 (4.32-7.75) | 4.76 (3.46-6.55) |
| **REGARDS-EHR** | | | | |
| EHR No / CMS No (N=1,756) | 209 (11.9) | 53.0 | Ref | Ref |
| EHR Yes / CMS No (N=212) | 55 (25.9) | 127.4 | 2.29 (1.72-3.06) | 1.79 (1.29-2.48) |
| EHR No / CMS Yes (N=130) | 49 (37.7) | 222.2 | 3.75 (2.75-5.11) | 3.18 (2.28-4.44) |
| EHR Yes / CMS Yes (N=119) | 66 (55.5) | 393.0 | 5.93 (4.52-7.78) | 4.69 (3.39-6.49) |
| | | | | |

**APPENDIX K Legend:** Total N = 2,217 adjudicated hospitalizations. Implicit/Explicit ICD-9 sepsis defined based on taxonomies of infection and organ dysfunction codes proposed by Angus, et al. in addition to explicit sepsis codes. CMS ICD-9 sepsis defined based on list of codes used in the CMS measure. REGARDS-EHR defined as infection event meeting modified EHR criteria proposed by Rhee, et al.[4] REGARDS-SOFA defined as infection event with ≥ 2 SOFA points across all organ systems (respiratory, cardiovascular, renal, hematological, hepatic, and neurological). REGARDS-qSOFA defined as infection events meeting ≥ 2 qSOFA criteria. *Includes in-hospital mortality. **Hazard ratios estimated using Cox proportional hazards models including each criteria individually as a binary variable, accounting for clustering by participant identifier. †Adjusted for year of event, age at the time of event, gender, race, income, education, smoking status (6 missing), alcohol use (38 missing), atrial fibrillation, chronic lung disease, chronic kidney disease, stroke, myocardial infarction, hypertension, dyslipidemia, diabetes, and obesity. SOFA = sepsis-related organ failure assessment; qSOFA = “quick” sepsis-related organ failure assessment; EHR = electronic health record. CMS = Centers for Medicare and Medicaid Services; ICD-9 = International Classification of Diseases, Ninth Revision; PY = person-years; CI = confidence interval.
